# Possible modulating functions of probiotic *Lactiplantibacillus plantarum* in particulate matter-associated pulmonary inflammation

**DOI:** 10.3389/fcimb.2023.1290914

**Published:** 2024-01-09

**Authors:** Nishant Gupta, N.S. Abd EL-Gawaad, Suhad Ali Osman Abdallah, M. Al-Dossari

**Affiliations:** ^1^ Medical Research and Development, River Engineering, Greater Noida, India; ^2^ Department of Physics, Faculty of Science, King Khalid University, Abha, Saudi Arabia; ^3^ Applied College, Khamis Mushait, King Khalid University, Abha, Saudi Arabia

**Keywords:** PM_2.5_-associated allergies, gut microbiota dysbiosis, gut-lung axis, pulmonary inflammation, probiotics supplementation, Nrf2 activation, alveolar cell protection, anti-inflammatory cytokines

## Abstract

Pulmonary disease represents a substantial global health burden. Increased air pollution, especially fine particulate matter (PM_2.5_) is the most concerned proportion of air pollutants to respiratory health. PM_2.5_ may carry or combine with other toxic allergens and heavy metals, resulting in serious respiratory allergies and anaphylactic reactions in the host. Available treatment options such as antihistamines, steroids, and avoiding allergens/dust/pollutants could be limited due to certain side effects and immense exposure to air pollutants, especially in most polluted countries. In this mini-review, we summarized how PM_2.5_ triggers respiratory hyperresponsiveness and inflammation, and the probiotic *Lactiplantibacillus plantarum* supplementation could minimize the risk of the same. *L. plantarum* may confer beneficial effects in PM_2.5_-associated pulmonary inflammation due to significant antioxidant potential. We discussed *L. plantarum’s* effect on PM_2.5_-induced reactive oxygen species (ROS), inflammatory cytokines, lipid peroxidation, and DNA damage. Available preclinical evidence shows *L. plantarum* induces gut-lung axis, SCFA, GABA, and other neurotransmitter signaling via gut microbiota modulation. SCFA signals are important in maintaining lung homeostasis and regulating intracellular defense mechanisms in alveolar cells. However, significant research is needed in this direction to contemplate *L. plantarum*’s therapeutic potential in pulmonary allergies.

## Graphical Abstract

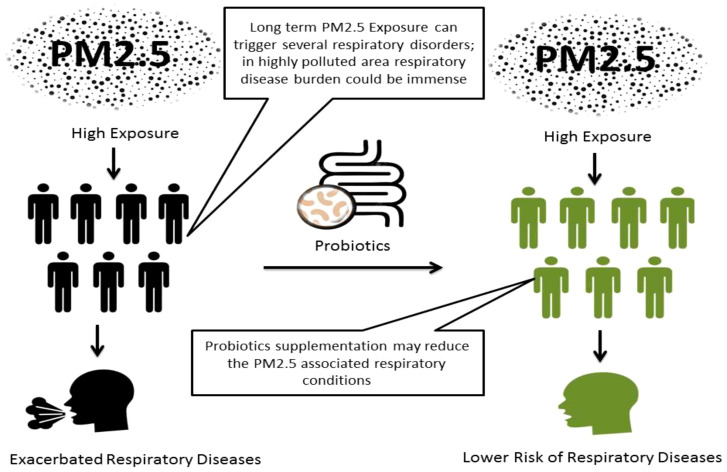

## 1 Introduction

Pulmonary diseases have been associated with substantial mortality and morbidity. Globally, around 545 million people live with chronic pulmonary conditions and may be vulnerable to premature mortality (Soriano et al., 2020). Asthma, chronic obstructive pulmonary disease (COPD), acute pulmonary infections, tuberculosis, and lung cancer remain the leading respiratory diseases (Ferkol and Schraufnagel, 2014). Inflammation is one of the most preliminary hallmarks associated with several pulmonary diseases. Airborne pollutants can aggravate airway defense mechanisms and induce the secretion of various primary innate defensive substances such as defensins, lysozymes, mucins, nitric oxide, and siderophores. Particulate matter size 2.5 μm (PM_2.5_) are most subtle air pollutant category, associated with the increasing burden of multiple health conditions including asthma, acute pulmonary distress, chronic obstructive pulmonary disease, and lung cancer. However, when epithelial cells and innate immune cells such as dendritic cells, eosinophils, macrophages, mast cells, monocytes, neutrophils, and natural killer cells eliminate airborne particles; inflammatory mediators cytokines such as interleukin-6 (IL-6), tumor necrosis factor (TNF) and reactive oxygen species (ROS) also produced along with (Li et al., 2018; Racanelli et al., 2018; Keulers et al., 2022). Recent findings indicated PM_2.5_’s potential role in COVID-19 pandemic and monkeypox exacerbation (Renard et al., 2022; Meo et al., 2023).

**Figure 1:**
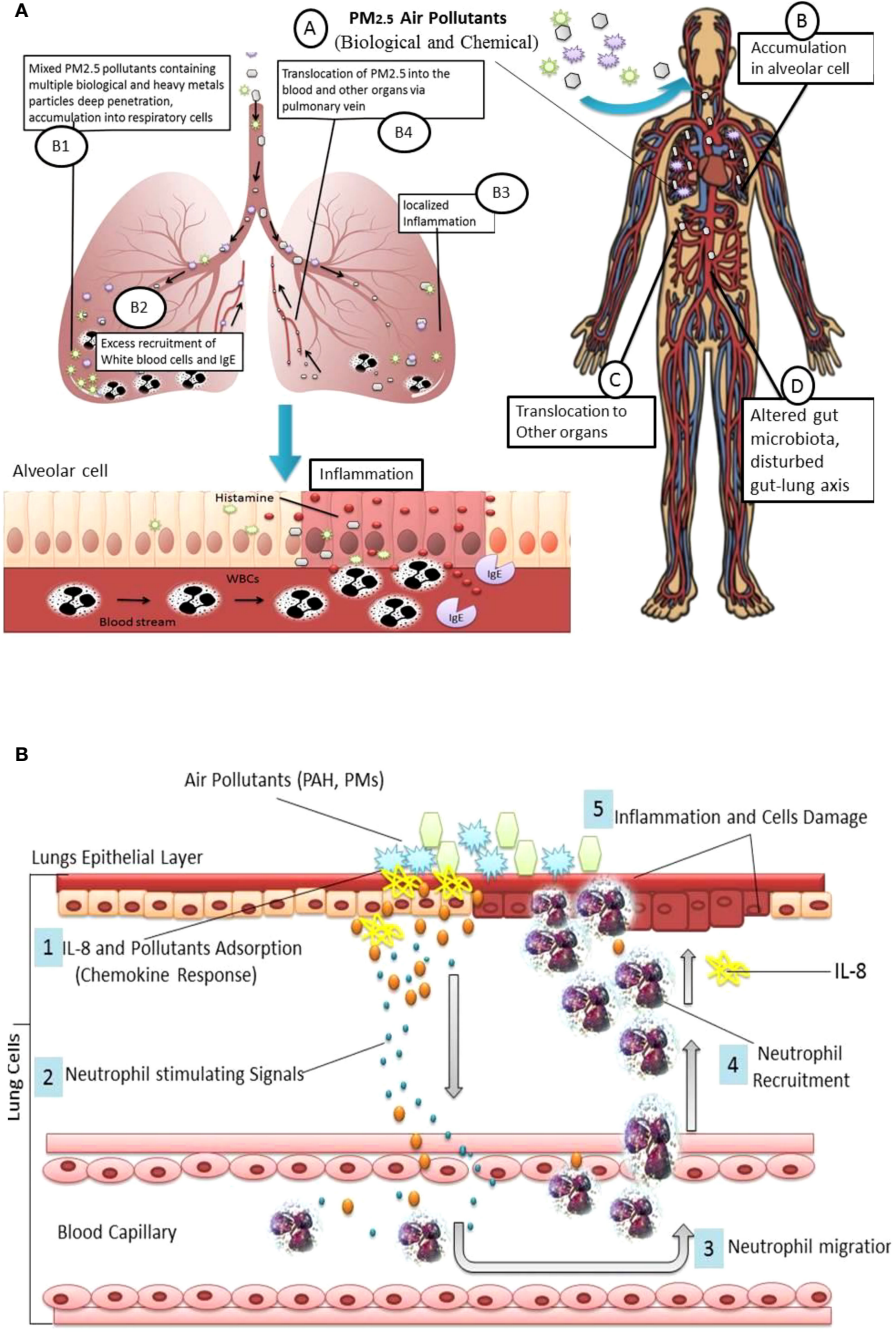
**(A)** PM_2.5_ derived lung inflammation and disturbed Gut-lung Axis: Inhalation of particulate pollutants (A); Interaction and accumulation PM_2.5_ with pulmonary epithelial cells (B); PM_2.5_ may contain a variety of pollutants such as heavy metals which absorb in alveolar cells (B1); accumulated pollutant trigger TLR associated inflammation, induced by excess production of ROS, change in mRNA expression and inflammatory cytokines, which facilitate WBCs and IgE recruitment (B2); excess recruitment of immune cells and histamine cause localized inflammation (B3); PM_2.5_ can translocate to other organs via pulmonary vein (B4); translocated PM_2.5_ may cause inflammation or damage to other organs (C) and altered gut microbiota composition, and disturbed Gut-lung and gut-organ specific (D). **(B)** Chemokines Response: PM_2.5_ and Polycyclic aromatic hydrocarbons (PAHs) triggered IL-8 and neutrophil deposition into lungs cells.

As per recent updates from the WHO, the world’s 99% population lives in areas where air quality has exceeded acceptable standards. Noticeably maximum proportion of this population belongs to less-developed countries (Gupta et al., 2022). High level of air pollutants such as PM_2.5_ may put the global population at substantial risk of respiratory disease (Lim et al., 2020). PM_2.5_ has a large surface area which may facilitate the carriage of other biological, chemical, and metallic hazardous compounds such as heavy metals. PM_2.5_ can accumulate deep into the lungs prompting local inflammation along with jeopardized lung functions, further, PM_2.5_ may relocate to other parts of the body through air exchange in the lungs, and trigger intracellular oxidative stress, mutagenicity and subsequently multiple medical anomalies, **Figure 1A** (Xing et al., 2016; Li et al., 2023). Especially, heavy metals in PM_2.5_ may cause high production of immunoglobulin E (IgE), IL-4, and IL-13 and immune dysregulation which trigger serious inflammatory responses (Zahedi et al., 2021). PM_2.5_-associated pulmonary allergies could be more serious compared to other allergens such as ovalbumin, pollens, and dust mites which usually involve type 1 T helper (Th1) and type 2 T helper (Th2) polarization. Exposure to high concentrations of PM_2.5_ may alter human microbiota profile and perturb gut-lungs (Gupta et al., 2022).

Pulmonary inflammation treatment is symptoms specific, usually exerting oral H1-antihistamines, immunotherapy, avoidance of environmental triggers, and even surgery in some rare cases. So far, steroids (corticosteroids) are frequently used as anti-inflammatory drugs employed for most of pulmonary complications including all types of asthma, allergic bronchopulmonary aspergillosis, hemangioma of trachea, and laryngotracheobronchitis. However, adverse effects and limitation of available treatment has been a major challenge so far (Sethi and Singhal, 2008; Meltzer et al., 2010) For instance, steroids have been frequently administered during COVID-19 pandemic, but effectiveness and the risk of routinely uses were skeptical (Waterer and Rello, 2020; Thakur et al., 2022). Severe immune suppression and related secondary infections such as black fungus incidences have also been observed during the delta variant of COVID-19 pandemic in India (Boretti, 2022). Moreover, corticosteroid resistance is a major concern among asthma and COPD patients (Barnes, 2013). Thus, the possibility of more treatment options could be important for the better management of respiratory ailments. Approaches such as phosphodiester (PDEs) enzyme inhibitors may be beneficial in chronic obstructive pulmonary disease (COPD) and asthma, especially Roflumilast-n-oxide has been approved as the add-on standard drug for the treatment of severe COPD. Other diverse organic compounds such as glutathione, tocopherol, flavonoids, essential oils volatile constituents may serve as natural antioxidants and reduce cellular oxidative stress by regulating ROS production which could be helpful in the management of subacute and chronic pulmonary oxidant stress (Christofidou-Solomidou and Muzykantov, 2006; Page and Spina, 2012; Horváth and Ács, 2015).

Growing evidence suggests the human microbial community such as gut and respiratory microbiota may play a pivotal role in lung homeostasis. Since lungs were previously considered sterile, recent studies showed the presence of a specific respiratory microbiome (Wypych et al., 2019; Gupta et al., 2022; Alsayed et al., 2023). Compelling outcomes showed that targeting gut microbiota in pulmonary ailments may be beneficial (Mahooti et al., 2020; Shahbazi et al., 2020). Preclinical and clinical trials suggest probiotic administration may reduce the risk of pulmonary infection (Wang et al., 2016). Mainly Lactobacillus spp, such as *Lactiplantibacillus plantarum* or *Lactobacillus plantarum* (LBP), role in pulmonary diseases has been discussed (Chong et al., 2019). However, the role of probiotics LBP in PM_2.5_ derived pulmonary allergies and inflammation is less understood as related research are scant so far. Thus, this short review discusses the putative role of LBP based probiotics in PM_2.5_ triggered airway inflammation and allergies.

## 2 PM_2.5_ induced airway inflammation

Pulmonary diseases have a strong correlation with fine particulate matter (PM_2.5_). Other air pollutants such as volatile organic compounds (VOCs), ozone, nitrogen oxide, sulfur dioxide, carbon monoxide, dioxins, polycyclic aromatic hydrocarbons (PAHs), heavy metals, biological antigens, dust may also trigger an immediate pulmonary anaphylactic reaction and poisoning (Duan and Tan, 2013; Manisalidis et al., 2020). But ultrafine particulate pollutants can be diffused in into the lungs alveolar and other regions. Despite physiological barriers like the nose itself which is a very efficient filter for ultrafine particles, many harmful air pollutants are able to infiltrate as not properly eliminated by mucociliary and macrophage-mediated mechanisms. Escaped air pollutants are potentially taken up by epithelial cells and may translocate to other organs via blood circulation and lymphatic system (Elder and Oberdörster, 2006). Most air pollution proportion is predominantly linked with particulate matter (PM) pollutants, ranging from 0.1 to 2.5 μm in diameter (PM_0.1_ and PM_2.5_). These pollutants can penetrate the pulmonary tract, accumulate within host cells, and induce cellular oxidative stress by increasing Reactive Oxygen Species (ROS) production in alveolar cells (Mazuryk et al., 2020). In addition, particulate pollutants can induce complex immune inflammatory response directly but molecular pathway of airway inflammation related lung damage has not been clearly understood so far (Esposito et al., 2014).

Pulmonary inflammation may be one of the preliminary responses associated with PM air pollutant inhalation. Long-term and excessive PM deposition may provoke hyper-responsiveness of the immune cells such as neutrophils and macrophages. Preclinical findings show pro-inflammatory chemokines such as IL-8 are mainly responsible for neutrophil recruitment into lung cells, **Figure 1B**. Similarly, macrophages may secrete chemokines into the epithelial lining fluid which may further interact with inhaled particulate matter and provoke localized deposition of neutrophils (Seagrave, 2008). Studies show that IL-8 is one of the important chemokines in air pollutant-induced airway inflammation (Watanabe et al., 2015; Kurai et al., 2018).

Thus, PM_2.5_ can be a serious environmental trigger, responsible for exacerbated immune response and inflammation of pulmonary cells, involving excess recruitment of white blood cells, immunoglobulin (IgE), and histamine release, **Figure 1A**. Aggregated lymphocytes lead inhibitions of cytokines INF-r and TGF-β; consequently IL-4, IL-5, IL-13, and IL-17a levels increased. PM_2.5_-induced inflammation may involve Toll-like receptors (TLR2/TLR4), increased production of reactive oxygen species (ROS), overproduction of inflammatory cytokines, lipid peroxidation, and DNA damage followed by cell damage.Lungs exposed to PM_2.5_ may undergo cellular oxidative damage and reduced level of antioxidants Glutathione/oxidized glutathione ratio and increased 3-nitrotyrosine, 4-hydroxynonenal levels increased (Wang et al., 2019). PM_2.5_ is also able to alter mRNA expression and inflammation-related (IL-6, IL-10, and IL-1β) genes in other parts of the human body such as the colon (Xie et al., 2022).

## 3 PM_2.5_ associated asthma and allergic rhinitis

**Figure 2:**
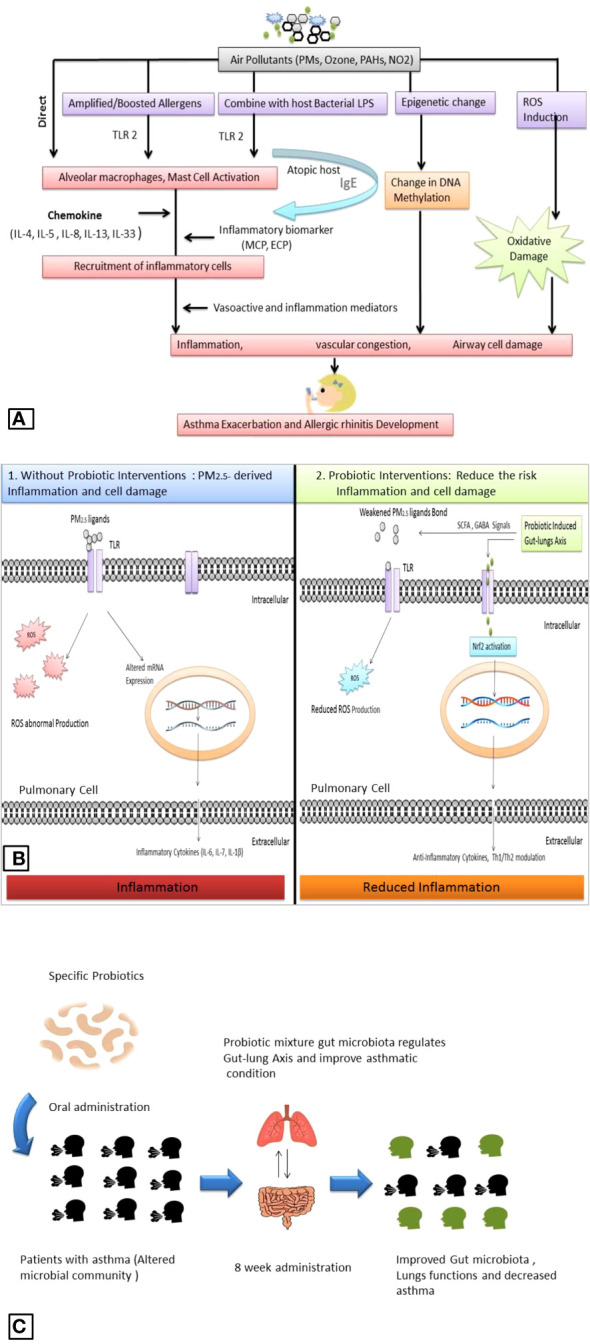
Air pollutant -associated asthma and allergic rhinitis exacerbation **(A)**. Comparative depiction of PM_2.5_ associated pulmonary inflammation and probiotics role: PM_2.5_ ligands bind with TLR and provoke ROS overproduction and alteration in mRNA expression which produces inflammatory cytokines (1); while probiotic supplementation induces the PM_2.5_ ligands and reduces the production of ROS, induce gut-lung axis induces intracellular defense system and activate Nrf2 which regulates mRNA expression and produces anti-inflammatory cytokines (2) **(B)**. Outcomes from recent clinical trials showed a probiotic positive effect on pulmonary function in asthmatic patients **(C)**.

Particulate pollutants can exacerbate asthma. There is a significant association between asthma and poor air quality (Tiotiu et al., 2020). In particular, asthmatic children are more susceptible to air pollution. The mechanisms of particulate matter and other air pollutant-induced asthma are less understood (Madaniyazi and Xerxes, 2021). Recent findings indicate PM may combine with other contaminants and allergens such as bacterial cell components lipopolysaccharide (LPS) and provoke pulmonary inflammatory response. Inflammatory factors such as TLRs 2 are in human alveolar macrophages activated by the combination of particulate pollutants and bacterial cell constituents (Esposito et al., 2014). Epigenetic alteration such as abnormal DNA methylation is also linked with asthma, and air pollutants such as PM_2.5_, and PAHs may cause alteration in DNA methylation on cytosine-guanine dinucleotide bond site (Rider and Carlsten, 2019; Bontinck et al., 2020). We have discussed that PM can also amplify allergens and trigger allergic rhinitis (Li et al., 2023). It is noticeable that PM_2.5_ does not act as an allergen but could interact with other allergic constituents like pollens, and microbial cells and accelerate their allergic capacity. For instance, ragweed and birch trees exposed to higher concentrations of air pollutants produce more allergens (Eguiluz-Gracia et al., 2020). In animal studies, it has been observed that particulate pollutants can also stimulate the other cytokines IL-33 and Th2-associated cytokines such as IL-4, IL-5, and IL-13 in alveolar cells. In allergy-sensitive individuals, specific development of IgE antibodies against pollutant-amplified allergens was observed. IgE antibodies associated with mast and other cells may cause a strong anaphylactic response during re-exposure to the same allergens and particulate pollutants resulting in several vasoactive and inflammation mediator agents such as histamine, prostaglandins, sulfidopeptide leukotrienes release from mast cells which cause immediate itching, runny nose, and vascular congestion (Naclerio et al., 2020), a potential mechanism given in **Figure 2**.

## 4 Probiotic effect on PM_2.5_ triggered Inflammation

The probiotic role in pulmonary ailments and inflammation has been discussed recently. Probiotics’ effect on PM_2.5_-associated pulmonary inflammation mainly involves gut microbiota modulation, which facilitates several protective pathways such as direct neutralization of particulate matter, oxidative stress removal, and induced cellular defense system. Probiotic bacteria, such as *Lactobacillus acidophilus*, and *Bifidobacterium lactis* have considerable capacity to bind the constituents of PM_2.5_ such as benzo(a)pyrene, combination with anti-oxidant vitamin C and E, and the anti-inflammatory docosahexaenoic acid alleviate PM_2.5_ antigenicity (Panebianco et al., 2019; Chiu et al., 2023). Mainly *Lactobacillus* spp. seems an important probiotic agent to eliminate pulmonary inflammation hallmarks. For instance, *Lactobacillus paracasei* HB89, *Lactobacillus casei* HY2782, and *Bifidobacterium lactis* HY8002 were able to reduce PM_2.5_-induced total white blood cell count, IL- 4, IL-5, IL-13, IL-17a level, ROS, activated superoxide dismutase and catalase activity in and in animal models, **Figure 2** (Lin et al., 2020; Nam et al., 2020).

## 5 *L. plantarum* supplements induced gut-lung axis

Compelling evidence suggests that gut microbiota plays a key role in regulating multi-axis relations to maintain human physiology including respiratory health. However, the composition of gut microbiota could be altered by atmospheric triggers such as particulate matter. Altered gut microbiota or microbial dysbiosis may also contribute to pulmonary diseases by interrupted gut-lung axis. The probiotic role has been the most promising agent to treat gut microbial dysbiosis and associated health risks. Interestingly, probiotics can directly facilitate the remediation of several pollutants such as heavy metals and Perfluorobutane sulfonate (Liu et al., 2020; Arun et al., 2021).

Recent clinical trials showed probiotics can improve pulmonary function in asthmatic patients. A study on 422 children, showed probiotic *Ligilactobacillus salivarius* LS01 (DSM 22775) and *Bifidobacterium breve* B632 (DSM 24706) mixture improved gut microbiota composition and reduced the number of asthma exacerbations when administered orally for 8 weeks, **Figure 2C** (Drago et al., 2022). Probiotic bacteria such as *L. plantarum* exert significant antioxidant potential and have shown promising outcomes against altered gut microbiota and oxidative stress. Cell constituents of *L. plantarum* such as exopolysaccharides (EPS) work as antioxidant agents and decrease the level of ROS directly (Angelin and Kavitha, 2020). Recent findings suggest *L. plantarum* can modulate the gut microbiota and maintain the gut as well as lung homeostasis. *L. plantarum* rebuilds the gut-organ-specific axis via SCFA and GABA signaling. SCFAs are the most important signal produced by the gut microbiota; SCFAs help to activate transcription factor nuclear factor erythroid 2-related factor 2 (Nrf2) which facilitates the intracellular antioxidant and anti-inflammatory pathway to minimize inflammatory hallmarks in damaged pulmonary cells (González-Bosch et al., 2021; Singh et al., 2022). Several strains of *L. plantarum* have shown promising effects against several airborne microbes, allergens, and air pollutants such as particulate matter, **Table 1**.

**Table 1:** Probiotic *L. plantaram* strains pulmonary protective effects against several airborne contaminants and air pollutants.

L. Plantaram strains	Type of Administration	Study model Animal/human	Inflammation Causing contaminant/pollutant	Key finding	References
*Lactobacillus plantarum* GCWB 1001	Oral	Mice	Diesel exhaust particulate matter	Prevent lung inflammation	Jin et al., 2020
*Lactobacillus plantarum-*CQPC11	Oral	Mice	Ovalbumin	Reduced airway hyperresponsiveness and prevention asthma	Lan et al., 2022
*Lactobacillus plantarum* NCC1107	Intragastrically/intranasally		Ovalbumin (allergen)	Reduced overall including eosinophilic lung inflammation	Pellaton et al., 2012
*Lactobacillus plantarum* NCIMB 8826	Oral	Mice	Influenza, Pulmonary Syncytial Virus	Minimize inflammation	Mai et al., 2020
*Lactobacillus plantarum* NCIMB8826	*In vitro* (Bacterial teichoic acid Mouse bone marrow-derived dendritic cell)	Mice	Aerosolized allergen	Reduced airway eosinophilia	Rigaux et al., 2009
*Lactobacillus plantarum* NC8	Orally administered mucosal vaccine	Chicken, mouse	Avian influenza viruses H9N2	hemagglutination-inhibition and induce robust T cell immune responses	Shi et al., 2016.

Modulated gut microbiota and its product SCFAs appear an important link between host immune defenses and gut flora (Sun et al., 2021). SCFAs’ role in lung health is important, presence of SCFAs in sputum confirms a connection between the gut and the lungs; thus, decreased levels of SCFAs may affect pulmonary immunity (Kotlyarov, 2022). Compromised SCFA may influence the overall homeostasis of the respiratory system and increase the risk of COPD, asthma, COVID-19, tuberculosis, and other infectious diseases (Ashique et al., 2022). A recent study shows SCFAs such as acetate, butyrate, and propionate administration may augment antiviral immunity and reduce virus load in rhinoviruses infected lung epithelial cell lines (Antunes et al., 2023). On the other hand, Gamma-aminobutyric acid type A receptors (GABAA-Rs) play a key role in neurodevelopment and neurotransmission in the central nervous system but also express by immune system. The activation of GABAA-Rs on innate immune system cells dendritic, macrophages, and natural killer (NK) cells may reduce inflammatory activities. GABA inhibits human immune cell secretion of many pro-inflammatory cytokines and chemokines associated with COVID-19 infection (Singh, and Rao, 2021; Tian et al., 2022). Thus *L. plantarum* supplementation associated induced SCFAs and GABA level may modulate the lungs homeostasis and immune response.

## 6 Conclusion and perspective

Add-on treatment options such as probiotic *L. plantarum* supplementation may be a helpful approach to managing the high risk of pulmonary ailments due to increasing air pollution. We have discussed that particulate matter has been the most concerned proportion of air pollutants associated with a substantial risk of respiratory ailments including asthma and COPD. In addition, PM_2.5_ amalgamation with allergens and heavy metals may trigger an immediate anaphylactic response. Reduction in atmospheric particulate matter and associated health risks has been an arduous challenge for years. Available treatment options are limited and show serious contraindications. In highly polluted, less developed countries, the risk of PM_2.5_-associated pulmonary ailments and allergies could be more serious due to less access to health facilities. Limited but important preclinical evidences show probiotic LBP supplementation can modulate the gut microbiota significantly and eliminate inflammatory hallmarks such as ROS, pro-inflammatory cytokines in alveolar cells and prevent airway inflammatory disorders like asthma, COPD, and rhinitis. We concluded that probiotic LBP supplementation could be an accessible and safe option to augment the existed treatment of pulmonary inflammation due to high PM_2.5_ exposure. However, considerable research is needed to understand the insight of probiotic *L. plantarum* in respiratory diseases.

## Author contributions

NG: Conceptualization, Data curation, Investigation, Writing – original draft. NA: Data curation, Formal analysis, Writing – review & editing. SA: Supervision, Visualization, Writing – review & editing. MA: Final Verification and Proofreading.
